# Markerless vs. Marker-Based Gait Analysis: A Proof of Concept Study

**DOI:** 10.3390/s22052011

**Published:** 2022-03-04

**Authors:** Matteo Moro, Giorgia Marchesi, Filip Hesse, Francesca Odone, Maura Casadio

**Affiliations:** 1 Department of Informatics, Bioengineering, Robotics and Systems Engineering (DIBRIS), University of Genova, 16145 Genova, Italy; giorgia.marchesi@edu.unige.it (G.M.); filip_hesse@yahoo.de (F.H.); francesca.odone@unige.it (F.O.); maura.casadio@unige.it (M.C.); 2 Machine Learning Genoa (MaLGa) Center, 16146 Genova, Italy; 3 Spinal Cord Italian Laboratory (S.C.I.L.), 17027 Pietra Ligure, Italy

**Keywords:** markerless, human motion analysis, gait analysis, computer vision, deep learning

## Abstract

The analysis of human gait is an important tool in medicine and rehabilitation to evaluate the effects and the progression of neurological diseases resulting in neuromotor disorders. In these fields, the gold standard techniques adopted to perform gait analysis rely on motion capture systems and markers. However, these systems present drawbacks: they are expensive, time consuming and they can affect the naturalness of the motion. For these reasons, in the last few years, considerable effort has been spent to study and implement markerless systems based on videography for gait analysis. Unfortunately, only few studies quantitatively compare the differences between markerless and marker-based systems in 3D settings. This work presented a new RGB video-based markerless system leveraging computer vision and deep learning to perform 3D gait analysis. These results were compared with those obtained by a marker-based motion capture system. To this end, we acquired simultaneously with the two systems a multimodal dataset of 16 people repeatedly walking in an indoor environment. With the two methods we obtained similar spatio-temporal parameters. The joint angles were comparable, except for a slight underestimation of the maximum flexion for ankle and knee angles. Taking together these results highlighted the possibility to adopt markerless technique for gait analysis.

## 1. Introduction

Gait analysis is a fundamental tool in medicine and rehabilitation [1]. It helps expert physicians to characterize and monitor motion patterns after orthopedic injuries and in people with neurological diseases, e.g., stroke, spinal cord injury, multiple sclerosis, or Parkinson [2]. Furthermore, gait analysis can be used to tailor appropriate and specific rehabilitation treatments. Quantitative assessments ensure repeatability and objectivity of the analysis, compared to visual observations only [3]. This kinematic quantification has been a major technical challenge for many years in the mid 90s [4].

Infrared marker-based motion capture systems (MoCap) have been developed to track continuous motion in 3D space [5]. Due to their high level of precision, infrared marker-based systems are considered the gold standard in modern gait analysis [6] and, in general, in accurate tracking of human motion. However, these approaches have limitations. First of all, they require many markers to be attached firmly to the body of the person. This process is time consuming and results in a cumbersome setup that can influence the naturalness of the motion [7]. These systems are also expensive and require skilled personnel to apply the markers correctly and to post-process the recorded data, making the overall analysis operator dependent [7]. Thus, the entire process requires many resources in terms of time and personnel. For these reasons, recently, many efforts have been made to study cheaper, faster, and simpler approaches to characterize human motion and, consequently, gait analysis [8]. Among the possible alternatives, systems based on wearable sensors (such as Inertial Measurements Units (IMU)) are less expensive, but suffer from the same issues of marker-based approaches.

In the last decades, deep learning algorithms have moved forward in solving computer vision problems [9]. In particular, recent advances on markerless pose estimation algorithms, based on computer vision and deep neural networks, are opening the possibility of adopting efficient methods for extracting motion information starting from common red-green-blue (RGB) video data [10]. This leads to the question of whether deep learning-based approaches can be adopted to analyze human motion in different domains and, specifically, if they can be adopted to perform accurate gait analysis for clinical applications [8]. Video-based techniques present many advantages with respect to marker-based systems. First of all, markerless video-based approaches are less expensive; they are not cumbersome and do not affect the naturalness of the motion, thus, they can be adopted to study human motion in an unconstrained environment. Lastly, they can be fully automatic and, hence, not operator dependent [6]. However, there are few studies that quantitatively compare the information extracted with video-based markerless techniques with those retrieved with gold standard marker-based systems [11,12,13]. As reported in the following section, most of them focus on 2D analysis, while for 3D analysis, to the best of our knowledge, there is still a lack of evidence related to the differences between video-based markerless and standard marker-based systems when describing meaningful kinematic variables and spatio-temporal parameters of human gait. In this work we aim at filling this gap by comparing both the spatio-temporal parameters and the joint angles changes during the gait cycle, computed from the keypoints extracted with these two techniques in 3D space.

Indeed, in this work, we defined an algorithm that, taking as inputs three RGB videos (acquired from 3 different viewpoints) and the calibration parameters, computes 3D keypoints positions. More precisely, our algorithm is composed by the following steps:

1. Keypoints detection in the image planes with a state-of-the-art Convolutional Neural Network (CNN): Pose ResNet-152 [14].
2. Keypoints refinement of the 2D detections, adopting Adafuse [15], that leverages epipolar geometry. In this step, also the weights of Pose ResNet-152 [14] are refined.
3. Keypoints’ trajectories temporal filtering to increase the spatio-temporal consistency.
4. 3D reconstruction: Combining the detected keypoints from the different viewpoints, we reconstructed the 3D positions of each keypoint following a geometric approach [16].

**Figure 1:**
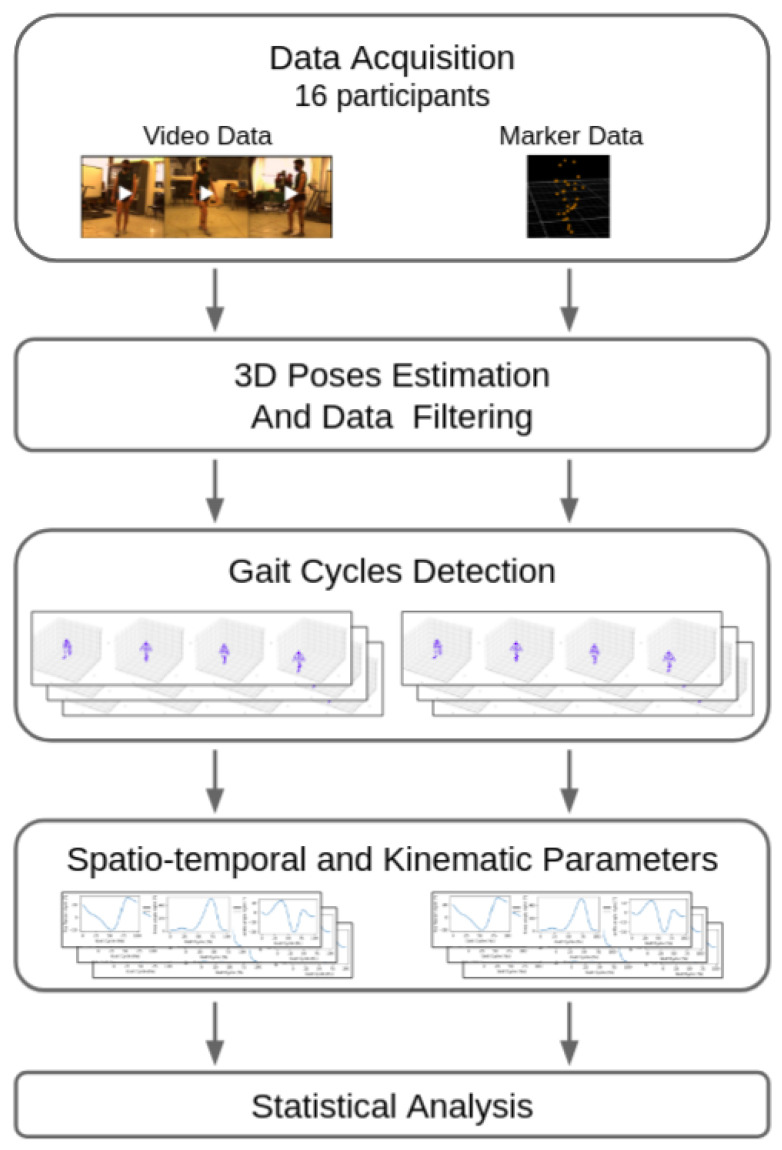
Summary of the workflow.

First, we trained our algorithm on the Human3.6M dataset [17]. Then, we used the trained model to extract the 3D keypoints positions from our acquired data. Starting from the 3D keypoints coordinates, we computed spatio-temporal and kinematic gait parameters. Then, we compared our method with the gold standard marker-based method. Figure 1 summarizes the main steps addressed in this work.

In this context, the main contributions of this work can be summarized as follows:

- Implementation of a video-based markerless pipeline for gait analysis. The pipeline takes as input RGB videos (multiple viewpoints of the same scene) and camera calibration parameters, computes the 3D keypoints following the algorithm summarized above, and gives as outputs the kinematic parameters usually computed in gait analysis.
- Comparison between marker and markerless systems. We tested the reliability and the stability of the implemented pipeline. To do that, we acquired the gait of 16 healthy subjects with both a marker-based system (*Optitrack*) and a multi-view RGB camera system. Then, by using a biomechanical model (OpenSim Software [18]), we computed the spatio-temporal and kinematic gait parameters [4] with data from both the gold standard motion capture system and our implemented markerless pipeline. Then, we compared the results from the two systems. Experimental results obtained in a preliminary study focusing on 2D data (single viewpoint) [19] provide initial evidence of the comparability of the two approaches.

The paper is organized as follow: In Section 2, related works that drives this research are presented; in Section 3 we present our sample and how we collected data; in Section 4, the 3D extraction’s procedure for both marker and markerless data are presented (Section 4.1 and Section 4.2, respectively); in Section 5, we presented the data filtering and the computation of spatio-temporal and kinematic parameters; in Section 6, the statistical tests used to compare the two approaches are presented; and in Section 7 and Section 8, we present our results and its related discussion.

## 2. Related Works

Many efforts have been spent in the last few years to implement and test video-based systems that are able to characterize human gait without using cumbersome and intrusive markers placed on the body skin. In this section, we present works that addressed this problem by following approaches that differ for: the dimensionality of the considered space (2D or 3D analysis), type of cameras, e.g., depth cameras (RGBD) or RGB cameras, and type of algorithms (deep learning or classical approaches).

Rodrigues et al. [20] developed a markerless multimodal motion capture system using multiple RGBD cameras and IMUs mounted to the lower limbs of the participants to estimate spatio-temporal parameters and joint angles. Corazza et al. [21] extracted the walking people’s silhouettes from 16 RGB camera views. These 2D silhouettes extracted from images recorded from different perspectives allowed the researchers to reconstruct the visual hull of the subject as a 3D model. By post-processing this model, the relevant joint angles could be determined. The authors could achieve a good performance determining the angles on the sagittal plane, however with larger errors on smaller angles, such as the knee adduction angle. Examples of similar approaches that used one or more RGB cameras and extracted silhouettes or used RGBD cameras can be found in [11,22,23,24,25].

Recently, due to the continuous progress in terms of accuracy and computational costs of pose estimation algorithms based on deep learning architecture, there is an increasing interest in the study of video-based systems for gait analysis. Kidzinski et al. [26] performed 2D gait analysis starting from the detection of keypoints in the image plane and, then, analyzing their trajectories extracting the joint angles and their changes on the gait cycle. They analyzed data from 1792 videos of 1026 patients with cerebral palsy. This approach has the potential to assess early symptoms of neurological disorders by using not expensive and commonly used technology. We followed a similar approach in Moro et al. [19] to investigate gait patterns in 10 stroke survivors. These works succeeded in performing a quantitative movement analysis using single camera videos in a stable way with results comparable to standard marker-based methods. Unfortunately, the 2D nature of the images limited the analysis to elevation angles [27] and to a subset of spatio-temporal parameters.

Vafadar et al. [28] performed markerless gait analysis by first reconstructing an accurate human pose in 3D from multiple camera views. They collected a gait-specific dataset composed by 31 participants, 22 with normal gait and 9 with pathological gait. The researchers recorded the gait of the participants with a standard marker-based system and with 4 RGB cameras. For 3D pose estimation, they relied on the approach proposed by [29]. They were successfully able to reconstruct the human pose while walking in 3D. However, they did not include in the detection keypoints on the feet and, consequently, they were not able to extract significant spatio-temporal parameters as the stride width and the ankle joint motion.

## 3. Dataset

A total of 16 unimpaired participants (6 females, mean age ± standard deviation: 27 ± 2 years old) without a known history of orthopaedic injuries or neurological diseases walked naturally in straight lines from one side of a room to the opposite. The path was 6 m long. Each participant performed 20 trials, 10 for each direction.

**Figure 2:**
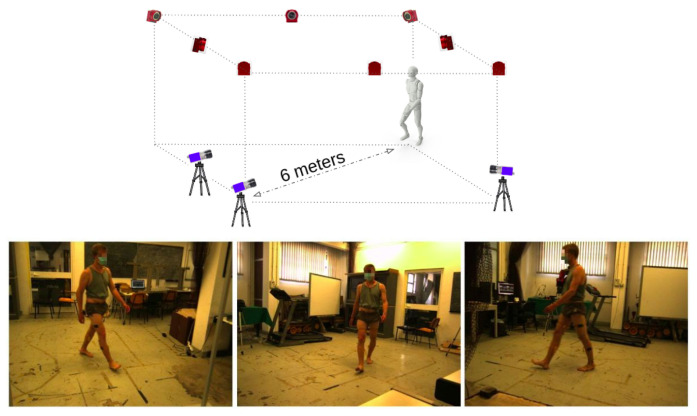
Setup adopted for data acquisition. The upper panel shows the sketch of the setup with the position of the 8 infrared (red) and 3 RGB (blue) cameras. The lower panel shows the three view points of the RGB cameras.

The setup for data acquisition (see Figure 2) included (i) a calibrated multi-view camera system consisting of 3 RGB Mako G125 GigE cameras with Sony ICX445 CCD sensor, resolution 1292 × 964, 30 frames per second (fps) for markerless analysis and (ii) a calibrated motion capture system, the Optitrack Flex 13 Motion Capture system, 1.3 MP, 56° Horizontal FOV, 46° Vertical FOV, 28 LEDs, 8.33 ms latency, with 8 cameras acquiring at 100 Hz. With the motion capture system, we acquired the 3D position of 22 infrared passive markers placed on the body of the participants following the Davis protocol [30]. RGB cameras calibration was performed according to Zhang’s method [31]. As a calibration pattern, we used a checkerboard with squares 40 × 40 mm. The calibration covered a volume of 6.5 × 2.5 × 2 m.

The study was conducted according to the guidelines of the Declaration of Helsinki, and approved by the Institutional Review Board of the Department of Informatics, Bioengineering, Robotics and Systems Engineering (DIBRIS), University of Genoa, Genova, Italy (protocol code CE DIBRIS-008/2020 approved on 18/05/2020). All the participants involved in the study signed an informed consent form.

## 4. 3D Keypoints

In this section, we present the processing for obtaining the 3D positions of meaningful keypoints. These steps are different for the marker-based and markerless approaches. More precisely, in the marker-based approach, we used the software Motive [32] to extract the 3D trajectories of the markers. In the markerless approach, we adapted the algorithm Adafuse [15] to detect and refine keypoints from the RGB videos. The two procedures are described in detail below.

### 4.1. Marker Data

**Figure 3:**
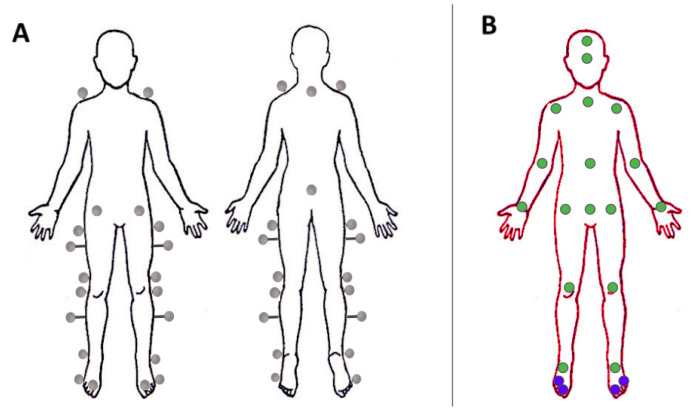
(**A**) Frontal and back views of the positions of the 22 markers positioned in this study according to the Davis protocol [30]. Specifically they were placed on the spinal process of C7 and on the spinal process of the sacrum (both visible in the back view) and bilaterally on: the acromion, the Anterior Superior Iliac Spine (ASIS), the greater trochanter, the middle between the greater trochanter and the lateral epicondyle of the femur (with bars 5 cm long), the lateral epicondyle of the femur, the fibula head, the middle between the fibula head and the lateral malleolus (with bars 5 cm long), the lateral malleolus, the first metatarsal phalangeal joint, and the fifth metatarsal phalangeal joint on the lateral aspect of the foot. (**B**) 2D keypoints (green and blue dots) considered in this work from the Human3.6 dataset. The two blue keypoints in each foot are highlighted because they are not included in [15] and we added them in our training.

The motion capture system reconstructed the trajectories of the markers in the 3D reference system, starting from 8 infrared cameras. To perform the motion analysis, we needed to add a feature matching and tracking step. The process of *sorting and tracking* the markers is a standard procedure performed after data acquisition with a motion capture system. The software Motive [32] provided with the Optitrack motion capture system automatically performed this procedure by applying a model of the human body, indicating the position of the markers (Figure 3A), defined by the user. However, in cases of markers occlusions or presence of disturbances as reflexes, this procedure required the manual intervention of the operator, resulting in a time consuming procedure. This workload emphasizes one drawback of the marker-based motion capture system. At the end of this process, we obtained 16 matrices $Pmarker^{j}$ with j=1,⋯,16 indicating the index for each participant, of shape $22\times 3\times M_{j}$ (22 representing the number of markers, 3 the ${\left(X,Y,Z\right)}_{m}$ markers’ coordinates in the 3D space in the markers reference system (${}_{m}$) and $M_{j}$ for the number of samples for the acquisition of the *j*-th participant).

### 4.2. Video Data

The RGB cameras produced video streams acquired from three views. To obtain the 3D points, we needed to detect semantic features in 2D and then triangulate them in 3D. The resulting 3D points were easily tracked, since each one of them was associated with a semantic meaning. Thus, the aim of this step was the detection of the 3D positions of keypoints that represent the analogous of markers and that can be adopted to perform gait analysis. To perform this step, it is possible to proceed in two different ways: (i) rely on a 2D pose estimator to detect the positions of the keypoints in the image planes of each viewpoint and then reconstruct the positions of each keypoint in the 3D space with a 3D reconstruction algorithm (e.g., [16])or (ii) rely directly on an end-to-end 3D pose estimator (see the review [8] for examples). We opted for the first option in order to have higher control in the number and in the position of the body keypoints detected in the image planes.

For this task we relied on AdaFuse [15]: A deep learning-based algorithm that allows one to accurately detect the positions of specific keypoints in the image plane and leverages classical stereo vision algorithms [16] to reconstruct the 3D positions of the detected keypoints. We selected Adafuse as it is one of the most recent (2021) and most precise [15] algorithms for 3D pose estimation. Its precision is due to the refinements in the image planes (2D) of the detected keypoints: It leverages epipolar geometry and on stereo vision algorithms to refine 2D detection. In this way, the 3D keypoints estimates are also more precise. In addition, the CNN (Convolutional Neural Network) for the 2D keypoints detection (2D backbone in the following sections) can be accurately selected based on the specific goal. In Section 4.2.1, we present and justify our choices.

Adafuse is mainly divided into the three following parts:

- A 2D pose estimator backbone (*Pose ResNet* [14]).
- A fusing deep learning architecture that refines the probability maps of each view generated in the first step. To accomplish this, the algorithm takes into account the information from neighboring views and it leverages epipolar geometry [16]. In this way it, is possible to enrich the information of each probability map at any point *x* by adding the information of the probability maps of its neighbor viewpoints.
- A geometric 3D reconstruction part that leverages the intrinsic and extrinsic camera parameters obtained during calibration.

#### 4.2.1. Adafuse Training

The pretrained 2D backbone models provided by AdaFuse authors [15] do not consider keypoints on the feet. Since these keypoints are necessary for gait analysis to compute the kinematic parameters related with the ankle joint (i.e., ankle dorsi-/plantar-flexion), we had to train the model with new data that also included keypoints on the feet. Moreover, to effectively train our model, we also needed a dataset with the 3D ground truth positions of each keypoint. The direct outputs of the AdaFuse algorithm are 2D probability maps ($U_{t}^{j,i,l}$) of each keypoint *l* for each input frame ($I_{t}^{j}$, at *t*-th time instant and for the *j*-th participant) for each viewpoint *i* (i={1,2,3}). The final 3D pose could be computed by geometric triangulation. This is true if the 2D ground truth positions of each keypoint are consistent between the different viewpoints. Unfortunately, this is not the case for most of the available datasets.

Among the public available datasets (well summarized in [11]), we relied on the Human3.6m dataset [17] because it included almost all the characteristics required by our analysis and described below. The Human3.6m dataset contains recordings of 11 professional actors (6 male, 5 female), performing in 17 different scenarios. Those scenarios are, for example discussion, smoking, taking photos, or walking. The actors wear natural clothes while having markers attached to their clothes (or skin, if the skin is visible). In total, the dataset includes over 3.6 million images with human poses. Each scene only shows one actor at a time, so this dataset is only suitable for single human pose estimation. The dataset includes both a multi-view RGB camera system (with 4 cameras) and a motion capture system with infrared cameras and 32 markers (see [17] for further details). Leveraging *Vicon’s* skeleton fitting procedure [33] and by applying forward kinematics, the Human3.6m dataset [17] provides both the 3D ground truth (i.e., the positions of the keypoints in the 3D space), and the 2D ground truth ( i.e., the positions of the keypoints projected into the 2D image planes) of the different viewpoints (see Figure 3B). The reader is referred to [17] for more details on how 3D and 2D ground truth were provided. Human3.6m was our best option, even if it presented drawbacks for our main goal. For example, the feet sometimes get rather blurry, mainly in the swing phase where one foot moves quickly. Additionally, the background carpet, under the lighting condition during the recordings, has color similar to the skin, so contrast decreases to a low level, where even for human observers, it would be hard to detect the keypoints precisely.

We fine tuned the Adafuse architecture in two steps:

1. **2D backbone**. We first focused on the 2D backbone network creating independent probability maps of the keypoints in Figure 3B for each separate input image and we fine tuned the Pose ResNet-152 [14] pretrained on the COCO dataset [34]. We did not train the network directly from scratch to reduce time and the amount of computational resources needed. We fine tuned the network by adopting a subset of the Human3.6m training images, i.e., we considered one image every 20 frames (for a total of 180,000 training images). This allowed us to have a training set with a reasonable number of frames sufficiently different from one another.
2. **Full architecture**. Then we focused on the fusing network which refines the maps with the help of the neighboring views. This second part of the AdaFuse architecture should not be trained separately (as mentioned in [15]), but jointly with the 2D backbone. Thus, we initialized the first part (2D backbone) with the weights obtained with the fine tuning described above and the fusion network with random weights. In this case, the inputs of the process are not just single images (as for the previous step), but a group of images representing the same time instant but coming from different viewpoints. Additionally, we input the calibration information for the group of images containing intrinsic and extrinsic parameters. These parameters are not used by the neural network itself, but in an immediate post-processing step which computes the 3D poses at the end. The target and output for the neural network is a group of probability maps corresponding to the input images. It is worth remembering here that the outputs of the full Adafuse process are just probability maps and not 3D points, however they are more precise than those from the 2D backbone because additional information from neighboring views is fused with the backbone prediction. The 3D pose is then computed via triangulation.

#### 4.2.2. Inference

**Figure 4:**
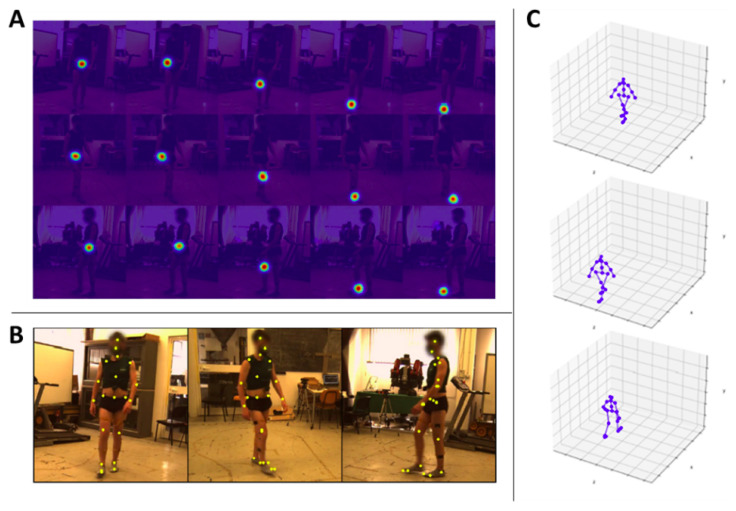
(**A**) Examples of the detected probability maps ($U_{t}^{j,i,l}$) for the *j*-th participant at a specific time instant *t*. The rows represent the 3 different viewpoints *i*. Each column represents a different keypoint *l* detected on the right leg (from left to right: hip, knee, heel, toe). (**B**) Examples of the detected keypoints (yellow dots) on the three views composing our dataset. (**C**) Examples of the final 3D skeleton of the video pre-processing.

We applied the model trained as described in Section 4.2.1 to our dataset for retrieving the 3D positions of the Human3.6m keypoints highlighted in Figure 3B. Since Pose ResNet-152 requires as input a bounding box also localizing the person in the image plane for each frame composing the videos, we relied on CenterNet [35], which is a state-of-the-art object detector, to create these bounding boxes for our dataset. Thus, we input to the model the 3 images coming from the 3 different viewpoints at the same time instant *t*, the bounding boxes, and the intrinsic and extrinsic parameters retrieved with cameras calibration. Firstly, we obtained the probability maps for different keypoints at the same time instant (Figure 4A for some examples) and then the final 2D locations of each keypoint (Figure 4B). At the end, the final output is a vector of shape 21×3 (21 keypoints with the corresponding ${\left(X,Y,Z\right)}_{v}$ coordinates in the 3D space in the camera reference system ${}_{v}$) with j=1,⋯,16 representing the number of videos (i.e., the number of participants) and $t=1,\cdots ,N_{j}$, which is the index for the number of frames composing the *j*-th video ($N_{j}$ is the total number of frame for the video of the *j*-th participant). At the end of this step, 16 matrices were left $Pmarkerless^{j}$ with a shape of $21\times 3\times N_{j}$ (see Figure 4C for examples of 3D poses).

### 4.3. Keypoints Detection Evaluation Metrics

To evaluate the accuracy of our trained model, we relied on two metrics usually adopted to evaluate the accuracy of 2D and 3D pose estimation algorithms.

For the evaluation of the 2D backbone, we relied on the Percentage of Correct Keypoints (PCK) [36]. Given the ground truth (as defined in [17] ) and the estimate position detected by our model of a certain keypoint *l* at the time instant *t* ($x_{t}^{l}$ and ${\tilde{x}}_{t}^{l}$, respectively), the PCK defines how close the estimate ${\tilde{x}}_{t}^{l}$ is with respect to the ground truth position $x_{t}^{l}$. In particular, ${\tilde{x}}_{t}^{l}$ is considered correctly detected if:

(1) $$\begin{array}{c} |{\tilde{x}}_{t}^{l}-x_{t}^{l}|<r_{thr} \end{array}$$

where $|{\tilde{x}}_{t}^{l}-x_{t}^{l}|$ represents the Euclidean distance between the estimate and the ground truth position of the keypoint *l*. This means that to be considered correctly detected, the estimate ${\tilde{x}}_{t}^{l}$ should fall inside a circle centered in the ground truth $x_{t}^{l}$ and with radius $r_{thr}$. In many works regarding 2D pose estimation algorithm [10], the PCK is computed considering $r_{thr}$ as a percentage of: (i) the torso diameter (usually the 20%); (ii) the head bone link (usually the 50%, PCKh@0.5 with *h* indicating the head bone link and @0.5 indicating a 50% threshold). In this work, we adopted PCKh@τ considering different thresholds τ, e.g., PCKh@0.5, PCKh@0.75, PCKh@1, corresponding 0.5, 0.75, and 1 time to the length of the head bone link.

On the other side, for the evaluation of the accuracy of the full process ending with the 3D reconstruction, we relied on the Mean Per Joint Position Error (MPJPE). The MPJPE is the most common metric to evaluate 3D estimates and it is defined for each keypoint as the mean euclidean distance in the 3D space between the estimated keypoint (${\tilde{x}}_{t}^{l}$) and the correspondent ground truth ($x_{t}^{l}$).

## 5. 3D Keypoints Trajectories Processing

The 3D trajectories of the keypoints extracted with marker-based ($Pmarker^{j}$) and markerless ($Pmarkerless^{j}$) systems were processed in the same way to extract quantitative parameters describing the gait of each participant.

### 5.1. Gait Cycle Detection

One gait cycle is defined as the period that starts with the heel strike (first instant when the heel hits the ground) of one foot and ends with the following heel strike of the same foot. A typical approach for automatic gait cycle detection in the absence of force platforms is to analyze the speed of the feet keypoints [37]. The cycle starts when the heel hits the ground; in this time instant, the speed of the heel is close to zero. It remains close to zero for the entire stance phase (the phase starting with the heel strike and ending when the foot leaves the ground) and it goes up in the swing phase (complementary to the stance phase). Then, the swing phase ends and the heel speed goes close to zero again. This first time instant where the speed is close to zero is the one representing the end of the current gait cycle and also the start of the following one.

For both the marker and the markerless approaches, we detected the start and the end of the gait cycle by following this procedure and considering the vertical component of the heel keypoint, low pass filtered with a Butterworth filter (4-th order, 3 Hz cut off frequency). We computed the derivative of the filtered vertical (*Y*) heel coordinates, obtaining the velocity profiles. Then, we computed the speed absolute value by combining the coordinates and we automatically detected the gait cycles following the considerations mentioned before. It is worth mentioning here that the 3 Hz cut off frequency filter was only used for gait cycle detection. To process the keypoints’ signals in later steps, we filtered the original raw signals as described in the following sections.

### 5.2. Spatio-Temporal Parameters and Joint Angles

The 3D coordinates trajectories of each keypoint during the gait cycles were low pass filtered (Butterworth, 4-th order, 12Hz cut off frequency) [4].

Starting from the heels’ markers trajectories, we extracted the spatio-temporal parameters that characterize the human gait. In particular, we computed the following parameters: (i) Stride length: the distance (in meters) walked during a gait cycle; (ii) Stride time: the time (in seconds) necessary to walk one gait cycle; (iii) Stance phase: percentage of the gait cycle during which the foot of interest is touching the ground; (iv) Swing phase: percentage of the cycle complementary to the stance phase, when the foot of interest is not touching the ground; (v) Stride width: the distance (in meters) between the right and the left foot across the cycle; and (vi) Speed: mean speed of the center of mass of the body during the cycle.

To estimate the joint angles during the gait cycle, we relied on the open source software Opensim [18]. Opensim is commonly adopted to estimate joint angles during gait analysis because it allows associating the detected keypoints/markers to human biomechanical skeleton models and analyze the kinematics and the relative muscular activation. In this work, we adopted the Rajagopal Model [38], a full body musculoskeletal model for dynamic simulations of human movements, widely used in gait analysis applications. In Opensim, two tools are specifically designed to solve our problem, *Scaling* and *Inverse Kinematics*. The first was adopted to scale a generic skeleton model to fit the input markers/keypoints data. The latter was used to simulate the motion of the skeleton and to estimate the joint angles for each gait cycle for each subject. Following the steps explained above, we extracted the joint angles for the central gait cycle of each trial (for a total of 20 gait cycle) for each participant involved in the study both with marker-based and markerless systems.

## 6. Statistical Analysis

To compare the time profile of the joint angles during the gait cycle obtained with the markerless and the marker-based gait analysis we used the statistical parametric mapping method, which is specifically designed for continuous field analysis [39] and is already used in similar applications in gait analysis [19]. In this study we applied this method to the 1D spatio-temporal variables describing the variations of the joint angles during the gait cycle by using the open source software spm1d [39]. Specifically, we performed a one dimensional paired *t*-tests. We tested the following null hypothesis: “There are no statistically significant differences between the gait angles obtained with our markerless approach and the gait angles obtained with the gold standard marker-based system”. The alpha level indicating the probability of incorrectly rejecting the null hypothesis was set at 0.05. Small values of *p* allow for the rejection of the null hypothesis. Indeed, if we obtain p>0.05, we can conclude that our statistical tests did not find significant differences between the gait angles obtained with our markerless approach and those obtained with the gold standard marker-based system. To follow a conservative approach, i.e., to maximize the possibility to find statistically significant differences between the results obtained with the two methods, we did not apply Bonferroni corrections. Notice that the application of corrections for multiple comparisons would decrease the probability to find significant differences between the single point curves. Furthermore, we compared the spatio-temporal parameters obtained with the two methods with a paired *t*-tests. Again, statistical significance was set for all statistics at the family-wise error rate of α=0.05.

## 7. Results

### 7.1. Architecture Evaluation

**Figure 5:**
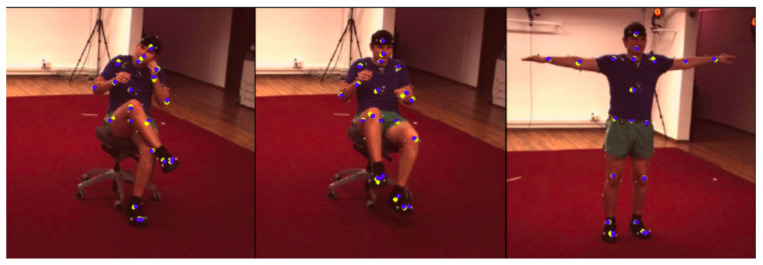
Examples of the keypoints detected with our model (yellow dots) with respect to the ground truth (blue dots).

To evaluate the accuracy of our trained 2D backbone, we computed the PCKh for each keypoint (see Figure 5 for a qualitative result). As threshold value $r_{thr}$, we selected a percentage of the head bone link for each participant (indicated by the *h* in PCKh). The following multiplication factors were chosen: 1 (PCKh@1), 0.75 (PCKh@0.75), and 0.5 (PCKh@0.5). Table 1 summarizes the obtained results.

The neural network indeed learned to detect also the new keypoints (toes and heels) with a high accuracy. The PCKh for these keypoints is comparable to the one of the others, and also to the results presented in other works (see for instance [15]).

To evaluate the accuracy of the full architecture, we computed the MPJPE across all the detected keypoints and obtained an error of 23.65 millimeters, again comparable to the one obtained in [15] (e.g., 19.5 millimeters on the same dataset, however with fewer keypoints–the feet were excluded) and also comparable with the error obtained in the best performing recent works about 3D pose estimation (between 19 and 30 millimeters) [40,41,42,43].

### 7.2. Joint Angles and Spatio-Temporal Parameters

We computed the spatio-temporal parameters described in the previous section for each gait cycle for every participant and compared the results obtained with the two different techniques. In Table 2, we report the mean and standard deviation across all the subjects. Note that parameters obtained with our markerless pipeline are similar to the ones extracted with the gold standard marker-based technique, as highlighted by the statistical comparisons: All *p*-values > 0.050, see Table 2 for more details.

**Figure 6:**
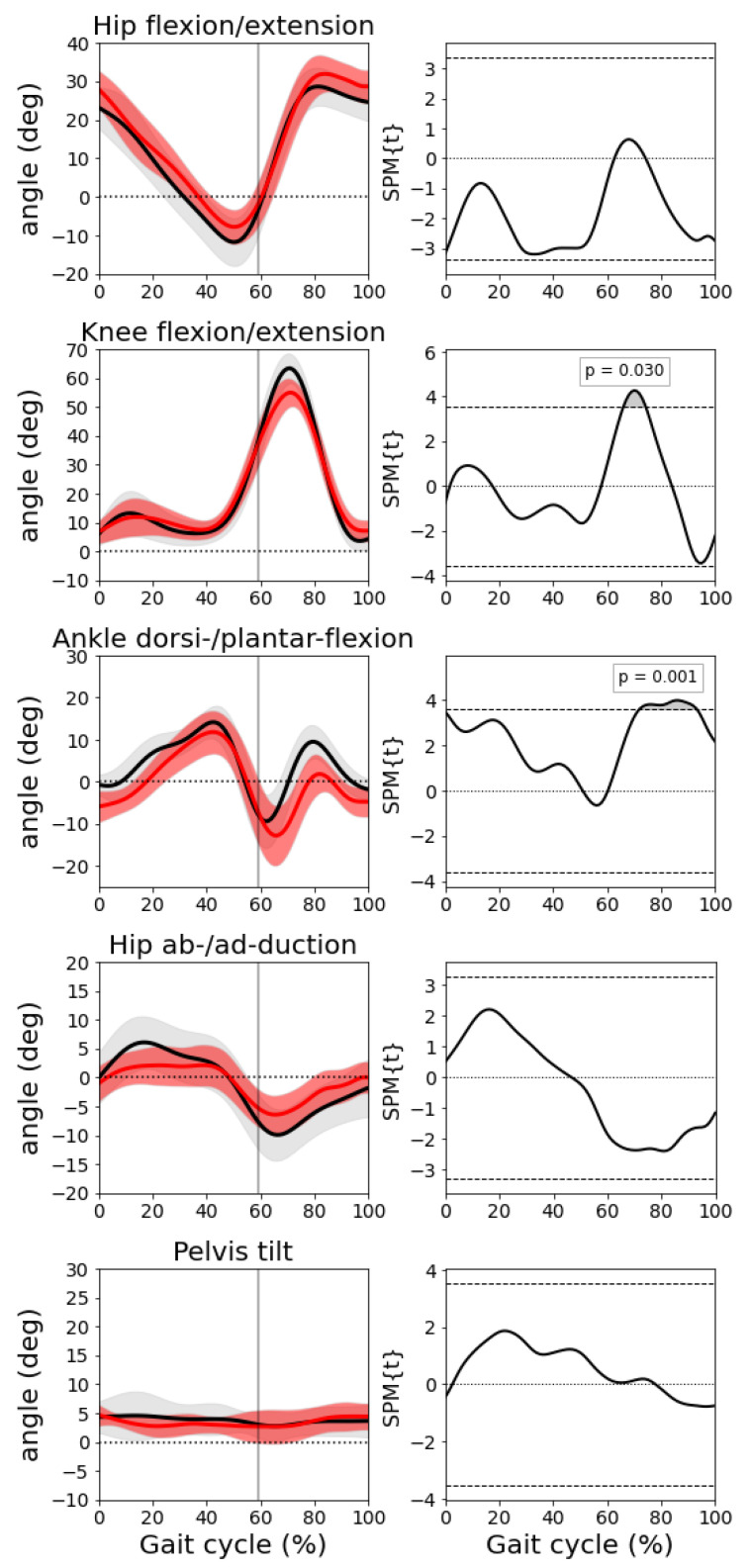
Left column: joint angles (mean and std). From top to bottom: hip flexion/extension, knee flexion/extension, ankle dorsi-/planta-flexion, hip ab-/ad-duction, and pelvis tilt. In black shows the results obtained with the marker-based system and in red shows the results with the markerless pipeline. Right column: results of the correspondent paired *t*-tests.

We compared the joint angles obtained by our markerless approach to those obtained with the marker-based method. We selected the following meaningful angles: hip flexion/extension, knee flexion/extension, ankle dorsi-/planta-flexion, hip ab-/ad-duction, and pelvis tilt. Figure 6 shows the mean and standard deviation of the angles previously mentioned across all the participants (black: marker-based, red: markerless) and the results of the paired *t*-tests. No statistical differences were found between the two techniques with the exception of a slight underestimation of the knee flexion and the ankle dorsiflexion angle between 70% and 80% of the gait cycle (during the swing phase, see gray areas in the paired *t*-testss in the right column of Figure 6 in correspondence of these two angles). Note that those statistical differences are not robust to multiple comparison, i.e., applying a Bonferroni correction the differences are not below the threshold for significance.

## 8. Discussion and Conclusions

This paper presents an approach for markerless gait analysis relying only on RGB video acquisition and leveraging computer vision and deep learning algorithms. Our approach presents the following advantages with respect to the gold standard marker-based methods:

1. It requires less expertise and has no bias introduced by any operators. In fact, while the operator during marker-based data acquisition needs to place markers carefully on the subjects skin in order to avoid biased results, our pipeline works fully automatically, and it is independent of any human performance;
2. It does not affect the naturalness of gait in any ways since it does not require cumbersome markers and sensors. Furthermore, it makes the data acquisition easier and faster because it is not necessary to place markers on the body skin;
3. It is less expensive and with a simpler setup and is easier to use outside laboratory environments, since it requires only RGB cameras.

Conversely, the results obtained with our markerless system present differences with respect to the ones obtained with the gold standard, especially during the swing phase in the maximum flexion of the knee and the ankle joint angles. These differences are statistically significant, however they appear to be small. Nonetheless, this limitation should be accounted and further investigated when adopting this markerless pipeline to detect and monitor abnormal motion patterns in people with orthopaedic injury or neurological diseases. If we focus on the errors related to the knee and the ankle joint angles during the swing phase, we can observe that they are mainly due to small errors in the detection of the feet keypoints. In fact, during the swing phase, the foot moves quickly and the image tends to become blurry, making it is difficult also for human beings to detect keypoints with high confidence. The immediate way to reduce the motion blur is to adopt RGB cameras with a higher temporal resolution, meaning a higher acquisition rate (fps). In this way, the motion blur will be reduced and, consequently, the detection error will also be lower.

Apart from inputting higher quality data to our pipeline, we can also improve the 2D backbone itself. In fact, the one adopted in this work and in AdaFuse [15] (Simple Baselines) is not the best performer according to multiple benchmarks. For example, the neural network HRNet [44] had been proven to provide better results on the Human3.6m dataset. Improving the accuracy of the detection will reduce the errors highlighted before.

In conclusion, the results suggest that the proposed markerless pipeline is a promising alternative to compute the marker-based system to most spatio-temporal and kinematic parameters. We highlighted also the limits of our pipeline and we presented possible solutions to overcome them in our future works.

## Figures and Tables

**Table 1:** Accuracy (%) of the 2D backbone, i.e., the percentage of corrected keypoints (PCKh) considering different threshold values: 1, 0.75, and 0.5 times the head bone link (PCKh@1, PCKh@0.75, and PCKh@0.5, respectively).

Keypoints	PCKh@1	PCKh@0.75	PCKh@0.5
head	96.3	95.8	95.2
root	96.6	95.6	94.8
nose	96.1	94.3	87.2
neck	96.1	89.3	77.2
right shoulder	93.4	87.4	66.7
right elbow	89.1	79.8	70.7
right wrist	85.5	78.6	67.8
left shoulder	95.2	88.9	72.7
left elbow	90.6	82.2	77.1
left wrist	85.0	78.7	70.0
belly	94.2	80.7	72.0
right hip	96.0	87.6	73.2
right knee	93.4	85.5	76.2
right foot1	91.6	79.7	61.4
right foot2	92.3	84.5	68.6
right foot3	89.2	77.3	63.0
left hip	95.8	85.1	72.1
left knee	92.4	79.9	66.7
left foot1	90.3	75.9	52.8
left foot2	91.7	83.4	67.7
left foot3	88.7	78.4	64.4

**Table 2:** Spatio-temporal parameters computed with marker-based and markerless systems, and statistical results of the comparison between the two methods (last row). We report the mean ± the standard deviation of each parameter. The stance and swing phases are reported in % with respect to the whole gait cycle; stride length and step width and expressed in meters (m); stride time in seconds (s); and the speed in meters per second (m/s).

	Stance Phase (%)	Swing Phase (%)	Stride Length (m)	Step Width (m)	Stride Time (s)	Speed (m/s)
**Marker**	59.2 ± 2.6	40.8 ± 2.6	1.35 ± 0.11	0.10 ± 0.02	1.13 ± 0.02	1.31 ± 0.10
**Markerless**	59.6 ± 3.1	40.4 ± 3.1	1.40 ± 0.21	0.12 ± 0.02	1.11 ± 0.04	1.35 ± 0.16
***p*** **-values**	0.644	0.644	0.474	0.132	0.291	0.341

## Data Availability

In our study, we relied on the public available dataset Human3.6m that you can find at http://vision.imar.ro/human3.6m/description.php (accessed on 30 December 2021).

## References

[1] Fritz N.E., Marasigan R.E.R., Calabresi P.A., Newsome S.D., Zackowski K.M. (2015). The impact of dynamic balance measures on walking performance in multiple sclerosis. Neurorehabilit. Neural Repair.

[2] di Biase L., Di Santo A., Caminiti M.L., De Liso A., Shah S.A., Ricci L., Di Lazzaro V. (2020). Gait analysis in Parkinson’s disease: An overview of the most accurate markers for diagnosis and symptoms monitoring. Sensors.

[3] Wren T.A., Tucker C.A., Rethlefsen S.A., Gorton G.E., Õunpuu S. (2020). Clinical efficacy of instrumented gait analysis: Systematic review 2020 update. Gait Posture.

[4] Whittle M.W. (2014). Gait Analysis: An Introduction.

[5] Cloete T., Scheffer C. Benchmarking of a full-body inertial motion capture system for clinical gait analysis. Proceedings of the 2008 30th Annual International Conference of the IEEE Engineering in Medicine and Biology Society.

[6] Colyer S.L., Evans M., Cosker D.P., Salo A.I. (2018). A review of the evolution of vision-based motion analysis and the integration of advanced computer vision methods towards developing a markerless system. Sport. Med.-Open.

[7] Carse B., Meadows B., Bowers R., Rowe P. (2013). Affordable clinical gait analysis: An assessment of the marker tracking accuracy of a new low-cost optical 3D motion analysis system. Physiotherapy.

[8] Desmarais Y., Mottet D., Slangen P., Montesinos P. (2021). A review of 3D human pose estimation algorithms for markerless motion capture. Comput. Vis. Image Underst..

[9] Voulodimos A., Doulamis N., Doulamis A., Protopapadakis E. (2018). Deep learning for computer vision: A brief review. Comput. Intell. Neurosci..

[10] Zheng C., Wu W., Yang T., Zhu S., Chen C., Liu R., Shen J., Kehtarnavaz N., Shah M. (2020). Deep learning-based human pose estimation: A survey. arXiv.

[11] Kwolek B., Michalczuk A., Krzeszowski T., Switonski A., Josinski H., Wojciechowski K. (2019). Calibrated and synchronized multi-view video and motion capture dataset for evaluation of gait recognition. Multimed. Tools Appl..

[12] Moro M., Casadio M., Mrotek L.A., Ranganathan R., Scheidt R., Odone F. On The Precision Of Markerless 3d Semantic Features: An Experimental Study On Violin Playing. Proceedings of the 2021 IEEE International Conference on Image Processing (ICIP).

[13] Needham L., Evans M., Cosker D.P., Wade L., McGuigan P.M., Bilzon J.L., Colyer S.L. (2021). The accuracy of several pose estimation methods for 3D joint centre localisation. Sci. Rep..

[14] Xiao B., Wu H., Wei Y. Simple baselines for human pose estimation and tracking. Proceedings of the European Conference on Computer Vision (ECCV).

[15] Zhang Z., Wang C., Qiu W., Qin W., Zeng W. (2021). AdaFuse: Adaptive Multiview Fusion for Accurate Human Pose Estimation in the Wild. Int. J. Comput. Vis..

[16] Hartley R., Zisserman A. (2003). Multiple View Geometry in Computer Vision.

[17] Ionescu C., Papava D., Olaru V., Sminchisescu C. (2013). Human3. 6m: Large scale datasets and predictive methods for 3d human sensing in natural environments. IEEE Trans. Pattern Anal. Mach. Intell..

[18] Delp S.L., Anderson F.C., Arnold A.S., Loan P., Habib A., John C.T., Guendelman E., Thelen D.G. (2007). OpenSim: Open-source software to create and analyze dynamic simulations of movement. IEEE Trans. Biomed. Eng..

[19] Moro M., Marchesi G., Odone F., Casadio M. Markerless gait analysis in stroke survivors based on computer vision and deep learning: A pilot study. Proceedings of the 35th Annual ACM Symposium on Applied Computing.

[20] Rodrigues T.B., Salgado D.P., Catháin C.Ó., O’Connor N., Murray N. (2020). Human gait assessment using a 3D marker-less multimodal motion capture system. Multimed. Tools Appl..

[21] Corazza S., Muendermann L., Chaudhari A., Demattio T., Cobelli C., Andriacchi T.P. (2006). A markerless motion capture system to study musculoskeletal biomechanics: Visual hull and simulated annealing approach. Ann. Biomed. Eng..

[22] Castelli A., Paolini G., Cereatti A., Della Croce U. (2015). A 2D markerless gait analysis methodology: Validation on healthy subjects. Comput. Math. Methods Med..

[23] Clark R.A., Bower K.J., Mentiplay B.F., Paterson K., Pua Y.H. (2013). Concurrent validity of the Microsoft Kinect for assessment of spatiotemporal gait variables. J. Biomech..

[24] Gabel M., Gilad-Bachrach R., Renshaw E., Schuster A. Full body gait analysis with Kinect. Proceedings of the 2012 Annual International Conference of the IEEE Engineering in Medicine and Biology Society.

[25] Saboune J., Charpillet F. (2007). Markerless human motion tracking from a single camera using interval particle filtering. Int. J. Artif. Intell. Tools.

[26] Kidziński Ł., Yang B., Hicks J.L., Rajagopal A., Delp S.L., Schwartz M.H. (2020). Deep neural networks enable quantitative movement analysis using single-camera videos. Nat. Commun..

[27] Borghese N.A., Bianchi L., Lacquaniti F. (1996). Kinematic determinants of human locomotion. J. Physiol..

[28] Vafadar S., Skalli W., Bonnet-Lebrun A., Khalifé M., Renaudin M., Hamza A., Gajny L. (2021). A novel dataset and deep learning-based approach for marker-less motion capture during gait. Gait Posture.

[29] Iskakov K., Burkov E., Lempitsky V., Malkov Y. Learnable triangulation of human pose. Proceedings of the IEEE/CVF International Conference on Computer Vision.

[30] Davis III R.B., Ounpuu S., Tyburski D., Gage J.R. (1991). A gait analysis data collection and reduction technique. Hum. Mov. Sci..

[31] Zhang Z. (2000). A flexible new technique for camera calibration. IEEE Trans. Pattern Anal. Mach. Intell..

[32] Motive: Optical Motion Capture Software. https://www.vicon.com/.

[33] Vicon. https://optitrack.com/software/motive/.

[34] Lin T.Y., Maire M., Belongie S., Hays J., Perona P., Ramanan D., Dollár P., Zitnick C.L. Microsoft coco: Common objects in context. Proceedings of the European Conference on Computer Vision.

[35] Zhou X., Wang D., Krähenbühl P. (2019). Objects as points. arXiv.

[36] Yang Y., Ramanan D. (2012). Articulated human detection with flexible mixtures of parts. IEEE Trans. Pattern Anal. Mach. Intell..

[37] O’Connor C.M., Thorpe S.K., O’Malley M.J., Vaughan C.L. (2007). Automatic detection of gait events using kinematic data. Gait Posture.

[38] Rajagopal A., Dembia C.L., DeMers M.S., Delp D.D., Hicks J.L., Delp S.L. (2016). Full-body musculoskeletal model for muscle-driven simulation of human gait. IEEE Trans. Biomed. Eng..

[39] Pataky T.C., Vanrenterghem J., Robinson M.A. (2015). Zero-vs. one-dimensional, parametric vs. non-parametric, and confidence interval vs. hypothesis testing procedures in one-dimensional biomechanical trajectory analysis. J. Biomech..

[40] Reddy N.D., Guigues L., Pishchulin L., Eledath J., Narasimhan S.G. TesseTrack: End-to-End Learnable Multi-Person Articulated 3D Pose Tracking. Proceedings of the IEEE/CVF Conference on Computer Vision and Pattern Recognition.

[41] He Y., Yan R., Fragkiadaki K., Yu S.I. Epipolar transformers. Proceedings of the IEEE/CVF Conference on Computer Vision and Pattern Recognition.

[42] Li W., Liu H., Ding R., Liu M., Wang P., Yang W. (2021). Exploiting Temporal Contexts with Strided Transformer for 3D Human Pose Estimation. arXiv.

[43] Shan W., Lu H., Wang S., Zhang X., Gao W. Improving Robustness and Accuracy via Relative Information Encoding in 3D Human Pose Estimation. Proceedings of the 29th ACM International Conference on Multimedia.

[44] Sun K., Xiao B., Liu D., Wang J. Deep high-resolution representation learning for human pose estimation. Proceedings of the IEEE/CVF Conference on Computer Vision and Pattern Recognition.

